# Supplementary material for: The Ubiquitination of Arrestin3 within the Nucleus Triggers the Nuclear Export of Mdm2, Which, in Turn, Mediates the Ubiquitination of GRK2 in the Cytosol
Source: Int J Mol Sci. 2024 Sep 6;25(17):9644. doi: 10.3390/ijms25179644 (PMC11395016; doi:10.3390/ijms25179644)
Supplement: Supplementary file 1 [file ijms-25-09644-s001.zip › ijms-3056009-supplementary.pdf]

The ubiquitination of arrestin3 within the nucleus triggers the nuclear export of Mdm2, which, in turn, mediates the ubiquitination of GRK2 in the cytosol

Dooti Kundu, Xiao Min, Xiaohan Zhang, Xinru Tian, Shujie Wang, Kyeong-Man Kim

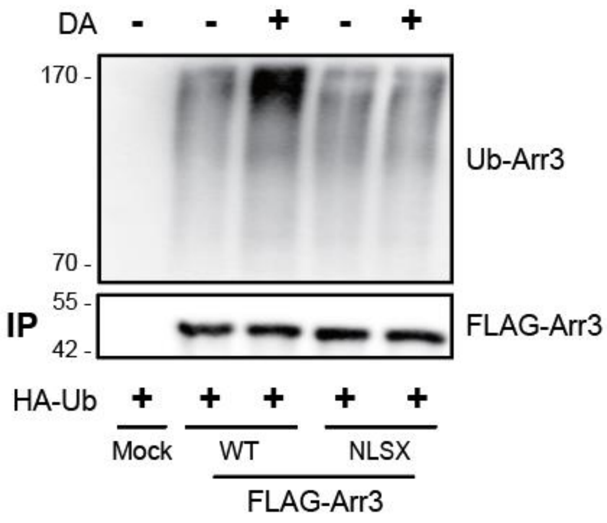

Figure S1. Comparison of D_2R-mediated ubiquitination of WT and NLSX-arrestin3. HEK-293 cells were transfected with D_2R (~1.7 pmol/mg protein), HA-Ub, and either a mock vector, FLAG-tagged WT-arrestin3, or FLAG-NLSX-arrestin3. Following a 2-minutes treatment with 10 μM dopamine, a ubiquitination assay was conducted as outlined in the experimental methods.
